# The Role of Thrombospondin 1 on Intestinal Inflammation and Carcinogenesis

**DOI:** 10.4137/bmi.s630

**Published:** 2008-03-28

**Authors:** Linda S. Gutierrez

**Affiliations:** Wilkes University, Wilkes-Barre, Pennsylvania U.S.A

**Keywords:** transforming growth factor beta 1, angiogenesis, ApcMin + mouse, CD36, inflammatory bowel disease

## Abstract

Crohn’s disease and ulcerative colitis are inflammatory bowel diseases (IBD) quite common in the United States and other Western countries. Patients suffering IBD are at greater risk of developing colorectal adenocarcinoma than the general population. Both, the adenomacarcinoma and the inflammation-carcinogenesis processes are characterized by active angiogenesis. Recent studies also have shown that anti-angiogenesis might be a novel therapeutic approach for IBD. Thrombospondin 1 (TSP1) is an extracellular protein well known for its anti-angiogenic properties. TSP1 also has key functions in inflammation, which is assumed to be the primary cause for carcinogenesis in IBD. This review is focused on the role of TSP1 in colorectal carcinogenesis. The therapeutic effects of TSP derived-peptides on inhibiting the inflammation-carcinogenesis progression are also discussed.

The role of inflammation in carcinogenesis has been broadly corroborated.(1) Granulocytes and monocytes produce growth factors and cytokines that stimulate tumor growth and induce changes in the stroma of tissues. These growth factors and cytokines also increase angiogenesis promoting tumor expansion and metastasis. Inflammatory cells produce reactive oxygen species that induce DNA damage.(2,3) DNA damage increases proliferation and induces cell genetic instability. All of these mechanisms will increase the chance of mutations and consequent malignancy.

There are many examples in which cancer is induced and promoted by inflammation. Colorectal carcinomas are among the best demonstrations for the crucial role of inflammation in cancer.(4) Chronic inflammation is a significant inductor of colorectal adenocarcinoma. The sequence hyperplasia-adenomacarcinoma for sporadic human colon cancer has been well studied.(5) However, the etiology and mechanisms involved in the transition from chronic inflammation-ulceration to dysplasiacarcinoma are not so well known.

Inflammatory bowel disease (IBD) such as Crohn’s disease and ulcerative colitis (UC) affect children and adults and they are recognized as premalignant disorders.(6,7) Sustained nitrosative/oxidative stress and DNA damage generated during chronic inflammation characterize IBD-related carcinogenesis.

UC-carcinogenesis could be a consequence of two main pathways: inflammation and hyperproliferation.(2) Previous experiments have shown that high levels of inflammation might trigger the initiation of dysplasia and the increased proliferation in the reactive epithelium could augment the chances for mutations.

The role of inflammation in colorectal carcinoma is demonstrated by the fact that non-steroidal anti-inflammatory drugs cause a dramatic involution of adenomas, the precursors of carcinomas.(8)

Angiogenesis is a landmark of cancer development and metastasis.(9) In addition there is a direct link between angiogenesis and inflammation.(10,11) Radical changes occur in the endothelium upon acute and chronic inflammation. Endothelial activation, dilation, increased permeability and leukocytic diapedesis are observed initially. Leukocytes and endothelial cells secrete high levels of interleukins, chemokines and growth factors enhancing the immune response and promoting angiogenesis.(10,11) The hyperproliferation and angiogenesis initiated by these factors as well as the DNA damage induced by leukocytic oxygen species all provide a foundation for carcinogenesis.(1,2)

The endothelium also secretes molecules that inhibit the immune response. As an example, factors involved in the protein C pathway are activated during inflammation and endothelial activation as well as leukocyte adhesion are both diminished.(12) These and other anti-inflammatory molecules might play a significant role in environments such as intestine and lung in which immune tolerance is critical.

Multiple evidence points to thrombospondin 1 (TSP1) as an inhibitor of angiogenesis and inflammation *in vitro* and *in vivo*.(13,14) In this review, the roles of TSP1 in colorectal carcinogenesis and inflammation are analyzed as well as the therapeutic effects of a TSP-derived peptide in IBD-related carcinogenesis.

These studies strongly suggest an important role of TSP1 in the mechanisms developing IBD and cancer. The use of TSP1 derived peptides could represent an innovative strategy to attenuate chronic inflammation and delay colorectal carcinogenesis.

## The Thrombospondins

The thrombospondins (TSP) include five calcium-binding extracellular glycoproteins.(13,14) TSP1 was the first to be identified and was detected in the α-granules released after platelet activation. TSP1 is also a component of the extracellular matrix.(16) The TSP family displays a series of epidermal growth factor-type repeats(TSR) called TSP type 2 repeats, and seven calcium binding repeats called TSP type 3 repeats.(16,17) Both, TSP1 and TSP2 have in addition three distinct repeats called TSP type 1 repeats.(15) These repeats (TSP structural repeats or TSR) are important in the functions of TSP1 and TSP2.(13–17)

The functions of TSP1 have been extensively studied in many laboratories. TSP1 is involved in the formation and resolution of a fibrin clot and binds to many proteins and proteases.(16) TSP1 interacts with heparan sulfates, proteoglycans, integrins, fibrinogen and fibronectin.(18) It also binds to components of the fibrinolytic system (plasminogen, urokinase and its inhibitor PAI-1)(19,20) and to cathepsin G and elastase.(21)

Numerous cell assays attribute functions to TSP1 in cell proliferation, apoptosis, angiogenesis inhibition, cell attachment and motility.(22) Most of these functions are shared with TSP2,(13–17) especially the inhibition of (EC) proliferation and angiogenesis. However, a motif KRFK between the first and second TSR binds and activates latent transforming growth factor beta 1 (TGFβ1); this site is not preserved in TSP2. Therefore TSP2 is unable to activate TGFβ1.(23,24) Through its binding to TGFβ1, TSP1 modulates cytokine response and secretion of other growth factors.(25)

Of great importance is the interaction of TSP1 with the scavenger receptor CD36. CD36 is expressed in endothelium (small vessels) and a variety of epithelial and stromal cells.(26) Some of the antiangiogenic functions of TSP1 are directly mediated by the binding of TSP1 with CD36.(27) TSP1 also has important functions in inflammation that are dependent of its binding with CD36.(28)

TSP1 is an inhibitor of EC proliferation.(29,30) However, its inhibitory effects on epithelial proliferation seem to be cell-dependent. TSP1 may modulate cell proliferation via TGFβ1 or by inhibiting angiogenesis.(31) The TGFβ1 family has direct inhibitory properties on epithelial proliferation. After its activation by integrins, proteases or by TSP1, TGFβ1 binds to its receptors: TGFβRI and TGFβRII. This process activates intracellular transcription factors called Smad 2 and Smad 3 that subsequently combine with Smad 4. This complex moves to the nucleus and activates regulatory genes. Genes involved in the cell cycle process such as the protooncogene c-myc, cyclins and p21 are regulated by this mechanism. Interestingly, mice deficient in Smad 2 develop more dysplasia and carcinomas after induction of chronic colitis.(32)

More recently, TSP1 has been reported to block the pro-angiogenic effects of nitric oxide (NO) in endothelial and vascular smooth muscle cells.(33) Low level of NO are anti-inflammatory, but in inflamed tissues the levels of NO increase and blood flow, vascular permeability and angiogenesis are enhanced.(11) By interacting with their receptors CD36 and CD47, TSP1 prevents the pro-angiogenic effects of NO and makes EC more susceptible to its inhibition.(33)

The anti-angiogenic effects of TSP1 may also be CD36 independent, it suppresses the cell cycle, increasing p21 and unphosphorylated retinoblastoma (Rb) in EC.(34) Many receptors interact with specific binding sites of TSP1. The sites with the anti-angiogenic functions have been recognized in the procollagen domain and type 1 repeats (TSR) sequences. TSR of TSP1 block vascular endothelial growth factor (VEGF)-induced migration of human umbilical vein endothelial cells (HUVEC) which lack CD36 and this inhibition is mediated by beta1 integrins.(35) TSP1 also interacts with other receptors involved in angiogenesis and inflammation such as integrin αvβ3 integrins,(36) proteoglycans and the integrin-associated protein CD47.(37) A component of the β3 integrin complex, CD47 is expressed in T cells and polymorphonuclear cells and it has a crucial role in T cell stimulation.(38,39)

By interacting with CD47, TSP1 might contribute to the quiescence state and self-tolerance in tissues such as intestine and lung. Upon binding with CD47, TSP1 promotes the activation of thymus-derived CD4^+^ CD25^+^ T regulatory cells (Tregs). These cells help to maintain self-tolerance and induce a suppressive phenotype.(40) Also, the disruption of the CD47/TSP1 pathway diminishes T cell apoptosis and delays leukocytic clearance in animal models.(41)

## TSP1 in Intestinal Inflammation

TSP1 increases neutrophil adhesion and migration. Also, TSP1 supports monocyte chemotaxis, haptotaxis, and diapedesis.(38,39) TSP1 might have a role in the clearance of inflammatory cells.(41) Inflammation and chronic conditions have been reported to show high expression of TSP1.(42)

The TSP1 null adult mice(43) have showed leukocytosis and abnormal inflammatory cell infiltrates in the lung. These infiltrates led to acute and chronic pneumonia with a consequent epithelial cell hyperplasia in the lungs. Lung architecture was disrupted with the formation of inflammatory pseudopolyps projecting into some of the airway lumens.

A feasible mechanism for these findings is that TSP1 activates the latent form of TGFβ1. The lack of TSP1 and consequent decrease of TGFβ1 activation would induce chronic active injury and increase of cytokines causing the epithelial hyperplasia. The role for TGFβ1 in inflammation seems to be the prevention of inappropriate responses to certain self-or environmental antigens.(44) When TGFβ1 signaling is blocked in T cells, inflammation is most severe at mucosal sites where exposure to environmental antigens is highest (gut and lung).(45)

TGFβ1 mediates intestinal healing and susceptibility to injury *in vitro* and *in vivo*.(44) Therefore, TGFβ1 activation is a crucial element in intestinal homeostasis. Dysregulated responses to the normal gut flora have been implicated in the pathogenesis of IBD. Mucosal T cells from patients with IBD express high levels of Smad7, an inhibitor of TGFβ1 signaling, suggesting that dysregulated TGFβ1 signaling might have a role in the pathogenesis of IBD.(44)

TGFβ1 downregulates epithelial cell proliferation *in vitro* and is a potent regulator of immune response.(45) By regulating the immune response TGFβ1 may reduce the formation of growth factors and free radicals, which promote cancer. The tgfb deficient mice show excessive inflammation and die in a short time.(46) TSP1 null mice show a similar but milder phenotype.

Normal murine and human intestinal mucosa express TSP1. In human colons, strong stain has been detected in the epithelium with progressive decrease in expression in adenomas and carcinomas.(47) Diffuse expression has been observed in the lamina propria and submucosa and strong cytoplasmic and membranous staining in platelets, endothelium, fibroblasts and granulocytes.

Treatment with Dextran Sulfate Sodium (DSS) induced a more severe colitis in TSP1^−/−^mice.(48) Focal inflammation was observed in colons after only two days of DSS treatment. This finding indicates that DSS induces an inflammatory response as an early effect, which was intensified by the deficiency of TSP1. TSP1^−/−^ mice displayed more bleeding, diarrhea and weight loss than controls as well as more extensive and deeper ulcerations.

A robust angiogenesis was a common feature in DSS-induced colitis in both genotypes. This process was present on the acute lesions as well as in regenerative areas. TSP1^−/−^ mice showed more angiogenesis than controls after only 2 days of DSS treatment.

Intense TSP1 expression was detected in the submucosa, among the inflammatory infiltrate in WT colons. Fibrin deposits and thrombus were also positive for TSP1(48). Upregulation of TSP1 has been detected in DSS induced-lesions using gene microarray technology(49). Gene profile also showed upregulation of well known factors involved in IBD such as tumor necrosis factor alpha (TNFα), TGFβ receptors 2 and 3 (TGFβR2 and TGFβR3) and interleukin 10 (IL-10).

TSP1 is considered a cell adhesion protein. It has also been described that bacterial cellular adherence could be promoted by proteins such TSP1.(50) In fact, TSP1 is a ligand protein for Clostridium difficile at the gastrointestinal epithelium(51) indicating a possible role of TSP1 on pathogenic interaction with host cells.

TSP1 might promote bacterial colonization and inflammation.(50) Studies investigating the impact of host TSP1 in the intestinal flora have been carried out.(52) Feces from TSP1^−/−^ mice contained a significantly lower density of fecal coliforms than feces from control mice, exposed or not to DSS. Density of lactobacilli did not vary significantly among samples. These data suggest that the lack of TSP1 in the colonic epithelium may affect the distribution of Escherichia coli (E. coli), possibly altering interactions between the bacteria and adhesin ligands in the epithelium. The mechanisms involved and the possible replacement of commensal E. coli by pathogenic strains during chronic colitis warrant further investigation.

## TSP1 on Intestinal Carcinogenesis

The functions of TSP1 on cancer are still controversial.(53) They may depend on the lack or presence of specific receptors or stromal ligands in specific tissue types. In some tumors TSP1 seems to promote tumor progression.(54–56) Other *in vivo* and clinical studies have shown an inverse correlation between TSP1 expression, malignancy and poorer prognosis.(57,58) When mice carrying the oncogene neu/erbB2 were crossed with TSP1 null mice, the number of mammary tumors and angiogenesis increased. Interestingly, mice overexpressing TSP1 showed tumor growth delay or inhibition.(59)

Clinical studies have reported a decrease in TSP1 protein expression in human colorectal carcinomas.(60,61) When TSP1^−/−^ mice were crossed with Apc^Min+/^, higher incidence in adenomas and carcinomas were observed.(62) Interestingly, no differences in vascular density were found between these mice and their littermates, suggesting TGFβ1 activation as the possible mechanism. Mice null for TGFβ1 and RAG2 (RAG^−/−^ mice are born without B and T cells) develop spontaneous adenomas and carcinomas in the colon.(63) These tumors were more frequently flat and highly dysplasic, similar to those in Apc^Min+/^ TSP1^−/−^ mice. TSP1 expression was significantly decreased in the stromal tissues of Apc^Min+/^ polyps. These adenomas also displayed significantly lower PCNA indexes for expression of proliferant cell nuclear antigen (PCNA) as compared to lesions developed in Apc^Min+/^ TSP1^−/−^ mice. These results indicate that higher proliferation and accelerated carcinogenesis are typical features in the lesions that arose in Apc^Min+/^ TSP1^−/−^ mice whose even smaller adenomas showed elevated PCNA indexes. Importantly, apoptotic indexes were unmistakably diminished on Apc^Min+/^ TSP1^−/−^ tumors, suggesting that TSP1 might also target tumor cells.

TGFβ1 decreases proliferation in colon carcinoma cells and a correlation between TSP1 and TGFβ1 expression has been found in human colon carcinomas.(64,65)

In a retrospective clinical study of more than 60 colorectal carcinomas, aberrant p53 expression was related with high expression of TSP1 and lymph node metastases.(66) Patients with primary colorectal cancer with low TSP1 expression, with or without detection of mp53 gene product, were more likely to have lymph node metastasis than patients with higher expression.

Using multiple and low doses of DSS, inflammation, apoptosis and key proteins in intestinal carcinogenesis such as p53 and βcatenin were evaluated in mice lacking TSP1.(67) In that study, endothelial apoptosis was decreased in TSP1^−/−^ colons tissues and secreted fas ligand was diminished compared to the levels detected in control mice. Foci of dysplasia, p53 and βcatenin nuclear expression were more frequently seen in TSP1^−/−^ mice. Although the grade of leukocyte infiltration was similar among the genotypes by the end of the study, higher levels of both, vascular endothelial growth factor (VEGF) and basic fibroblast growth factor (bFGF) were secreted by TSP1^−/−^ colons. These data support the idea that angiogenesis is initiated by chronic inflammation but even if this condition is resolved, DNA damage and changes in the epithelium will perpetuate the angiogenesis cycle and trigger carcinogenesis.

## Therapeutics Effects of TSP1 Derived Peptides in Intestinal Inflammation and Carcinogenesis

In recent studies, anti-angiogenic compounds have proved to be effective in IBD models.(68) TSP1 in combination with irinotecan inhibited tumor growth in a model of colon cancer xenograft.(69) ABT-510, a peptide derived from TSP1 has been studied in clinical studies (phase I and II for lymphoma and osteosarcoma). ABT-510 is a truncated molecule (recombinant human peptide) having most of the carboxyl terminal sequence removed with preservation of only a short portion of the N-terminal remaining.

ABT-510 has been found effective as therapy in brain tumors. In these studies, ABT-510 induced apoptosis in tumor cells and diminished angiogenesis and proliferation.(70) In the Apc^Min+/^model, ABT-510 administration using osmotic mini-pumps significantly diminished the number of adenomas compared with mice controls.(71) Angiocidin a peptide with TSP1 binding activity has been effective in a human colorectal xenograft model. In that study, the anti-tumor activity of this molecule is specifically mediated in its TSP1 binding site.(72,73)

In the DSS model of induced colitis, ABT-510 consistently diminished angiogenesis and bleeding in treated mice.(48) This peptide is an analogue of the heptapeptide of TSP1 that interacts with CD36; thus, induction of endothelial apoptosis and decreased angiogenesis were expected. Surprisingly, mice treated with ABT-510 also displayed less colonic inflammation. CD36 activation could have consequences in the immune response, favoring leukocyte apoptosis and clearance.(74) In addition, ABT-510 has been proved to induce caspase activation and apoptosis independently of its interaction with CD36.(75)

An important mechanism could be the TSP1/NO interaction. Decreasing the levels of NO by TSP1 will also inhibit leukocyte activation and angiogenesis.(33) In addition, diminished endothelial proliferation and induction of apoptosis by ABT-510 could restore the quiescent “resting” state of the endothelium. An inactive endothelium will sequester fewer leukocytes and will secrete reduced levels of pro-angiogenic factors.(11)

In conclusion, the relevance of TSP1 on colorectal inflammation and carcinogenesis has been reviewed emphasizing recent data. TSP1 seems to play an important pathological function in these processes acting by multiple pathways. The study of these signaling mechanisms could have a significant impact on future therapeutic strategies for IBD and colorectal cancer.

## Figures and Tables

**Figure 1:**
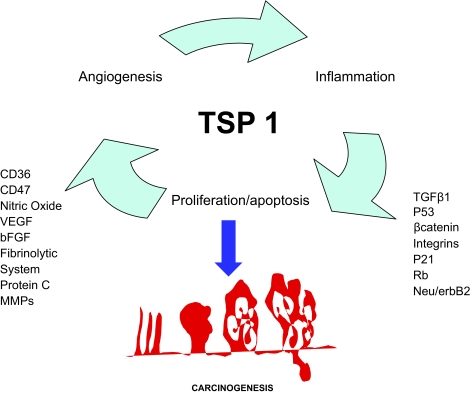
Schematic overview of main proteins and agents interacting directly or indirectly with TSP1 on angiogenesis, cell cycle and inflammation. All of them might regulate these mechanisms and consequently the development of colorectal cancer.
